# Passive transfer of Ad26.COV2.S-elicited IgG from humans attenuates SARS-CoV-2 disease in hamsters

**DOI:** 10.1038/s41541-021-00427-z

**Published:** 2022-01-10

**Authors:** Lisa H. Tostanoski, Abishek Chandrashekar, Shivani Patel, Jingyou Yu, Catherine Jacob-Dolan, Aiquan Chang, Olivia C. Powers, Daniel Sellers, Sarah Gardner, Julia Barrett, Owen Sanborn, Kathryn E. Stephenson, Jessica L. Ansel, Kate Jaegle, Michael S. Seaman, Maciel Porto, Megan Lok, Brittany Spence, Kathleen Cayer, Danielle Nase, Shaikim Holman, Heath Bradette, Swagata Kar, Hanne Andersen, Mark G. Lewis, Freek Cox, Jeroen T. B. M. Tolboom, Anne Marit de Groot, Dirk Heerwegh, Mathieu Le Gars, Jerald Sadoff, Frank Wegmann, Roland C. Zahn, Hanneke Schuitemaker, Dan H. Barouch

**Affiliations:** 1 Center for Virology and Vaccine Research, Beth Israel Deaconess Medical Center, Harvard Medical School, Boston, MA USA; 2 Harvard Medical School, Boston, MA USA; 3 BIOQUAL, Inc., Rockville, MD USA; 4 Janssen Vaccines & Prevention, Leiden, The Netherlands; 5 Janssen Research & Development, Beerse, Belgium; 6 Ragon Institute of MGH, MIT and Harvard, Cambridge, MA USA; 7 Massachusetts Consortium on Pathogen Readiness, Boston, MA USA

**Keywords:** Vaccines, Vaccines

## Abstract

SARS-CoV-2 Spike-specific binding and neutralizing antibodies, elicited either by natural infection or vaccination, have emerged as potential correlates of protection. An important question, however, is whether vaccine-elicited antibodies in humans provide direct, functional protection from SARS-CoV-2 infection and disease. In this study, we explored directly the protective efficacy of human antibodies elicited by Ad26.COV2.S vaccination by adoptive transfer studies. IgG from plasma of Ad26.COV2.S vaccinated individuals was purified and transferred into naïve golden Syrian hamster recipients, followed by intra-nasal challenge of the hamsters with SARS-CoV-2. IgG purified from Ad26.COV2.S-vaccinated individuals provided dose-dependent protection in the recipient hamsters from weight loss following challenge. In contrast, IgG purified from placebo recipients provided no protection in this adoptive transfer model. Attenuation of weight loss correlated with binding and neutralizing antibody titers of the passively transferred IgG. This study suggests that Ad26.COV2.S-elicited antibodies in humans are mechanistically involved in protection against SARS-CoV-2.

## Introduction

**Fig. 1:**
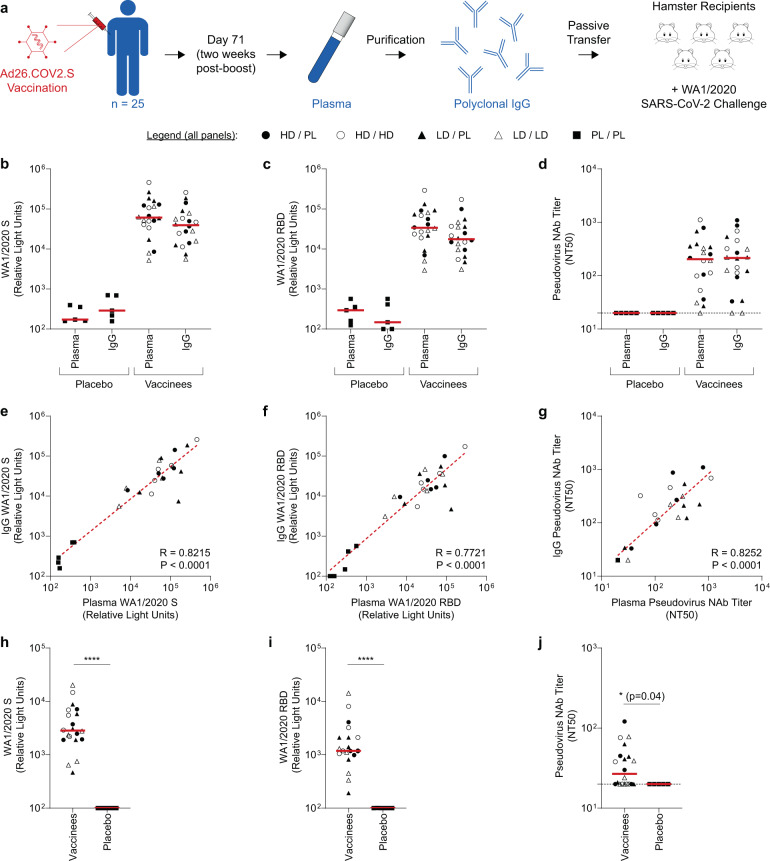
Ad26.COV2.S vaccine elicits binding and neutralizing antibodies in plasma and purified IgG from human vaccine recipients. **a** Schematic of the experimental design. Particpants in the COV1001 clinical trial were vaccinated with Ad26.COV2.S or placebo control. On day 71, plasma samples were collected and total IgG was purified. IgG was then transferred into naïve hamster recipients, followed by intra-nasal challenge with WA1/2020 SARS-CoV-2. **b**–**d** Binding and neutralizing activity was quantified in human plasma samples and corresponding IgG purified from plasma samples from either placebo recipients (*N* = 5) or recipients of the Ad26.COV2.S vaccine at the indicated dosing regimens (*N* = 20). Binding antibody responses were quantified via electrochemiluminescent assay (ECLA) specific for (**b**) WA1/2020 SARS-CoV-2 spike (S) protein and (**c**) WA1/2020 SARS-CoV-2 receptor binding domain (RBD) protein. **d** Similarly, neutralizing antibody titers were quantified using a pseudovirus neutralization assay. Data displated indicate the 50% neutralization titer (NT50) of a WA1/2020 SARS-CoV-2 S-expressing pseudovirus. In panels a-c, each data point displayed corresponds to one clinical trial study participant and red lines indicate the group medians. **e**–**g** Correlation of (**e**) S binding, (**f**) RBD binding, and (**g**) pseudovirus neutralizing antibody titers in pre-purification human plasma and corresponding purified IgG. Each data point displayed corresponds to one study participant. Statistics indicate the results of Spearman correlation analysis. **h**–**j** Purified IgG from vaccinees or placebo recipients was transferred to groups of naïve hamsters (*n* = 4–5 hamsters/study participant) via intraperitoneal injection. One day post-transfer, hamster serum was collected and analyzed for (**h**) S binding, (**i**) RBD binding, or (**j**) pseudovirus neutralizing antibody titers. Data points displayed in panels **h**–**j** correspond to the median titer of each group of *n* = 4–5 hamsters that corresponds to one clinical trial study participant. Horizontal red lines indicate medians of groups corresponding to vaccinees or placebo recipients. Statistics displayed are the results of two-sided Mann–Whitney tests. (****P ≤ 0.0001).

Pre-clinical studies of candidate SARS-CoV-2 vaccines support spike-specific binding and neutralizing antibody titers as a strong immune correlate of protection^1–4^. The importance of antibodies in the control or attenuation of COVID-19 is also highlighted by the success of monoclonal antibodies with potent neutralizing activity as prophylactic or therapeutic interventions^5–7^. In addition, we have previously reported that passive transfer of purified polyclonal IgG from convalescent non-human primates (NHPs) confers protection in the NHP model of SARS-CoV-2 infection in a dose-dependent manner^8^ and that spike binding and neutralizing antibodies correlate with protection in the NHP model. However, direct evidence of the protective efficacy of vaccine-elicited antibodies from humans remains unexplored. Pre-clinical^1,3,4,9,10^ and clinical studies^11–14^ of the Ad26.COV2.S vaccine demonstrated strong immunogenicity, as well as robust protection from severe clinical disease and hospitalization in large-scale efficacy trials^14^. We therefore sought to characterize the function of vaccine-elicited antibodies from humans in adoptive transfer studies using the SARS-CoV-2 hamster model^10^. Polyclonal IgG was purified from participants in cohort 1b from the Phase 1/2a trial COV1001 (NCT04436276) of the Ad26.COV2.S vaccine^11^ and administered to naïve hamsters (Fig. 1a). One day post-transfer, hamsters were challenged with WA1/2020 SARS-CoV-2 and monitored for body weight loss as a metric of clinical disease^10^ to examine the potential for vaccine-elicited antibodies to attentuate disease severity.

## Results

### Purification and characterization of vaccine-elicited human IgG

In COV1001, participants in cohort 1b were randomly allocated to one of five experimental groups (*n* = 5/group):^12^ (i) 1 × 10^11^ viral particles (vp) of Ad26.COV2.S (high-dose (HD)) on day 1 and day 57 (high-dose/high-dose (HD/HD)), (ii) high-dose on day 1 and placebo on day 57 (high-dose/placebo (HD/PL)), (iii) 5 × 10^10^ vp of Ad26.COV2.S (the standard clinical dose of authorized vaccine, indicated here with low-dose (LD)) on day 1 and day 57 (low-dose/low-dose (LD/LD)), (iv) low-dose on day 1 and placebo on day 57 (low-dose/placebo (LD/PL)), or (v) placebo on day 1 and day 57 (placebo/placebo (PL/PL)) (Supplementary Table 1). On day 71, two weeks after the second administration of either placebo or vaccine, plasma was collected from all participants and total IgG was purified from plasma samples. Pre-purification plasma and post-purification IgG stocks, normalized to 10 mg ml^−1^, were characterized using both binding and pseudovirus neutralization assays^15^.

As expected, both plasma and purified IgG from placebo recipients exhibited minimal binding to WA1/2020 spike (S) protein (Fig. 1b) or WA1/2020 receptor binding domain (RBD) protein (Fig. 1c). In contrast, samples from vaccine recipients exhibited high binding activity, with similar median titer values observed in plasma (S: 6.1 × 10^4^; RBD: 3.4 × 10^4^) and IgG (S: 3.9 × 10^4^; RBD: 1.8 × 10^4^). Further, in a neutraliziation assay, using a pseudovirus expressing WA1/2020 S, no neutralizing activity was observed in IgG or plasma from placebo recipients (Fig. 1d). In vaccinees, however, detectable 50% neutralizing titers (NT50) were observed in 19/20 plasma samples and 18/20 purified IgG stocks. Pre-purification plasma and purified IgG exhibited similar median NT50 values of 204 and 215, respectively. Further, a strong positive correlation was observed between pre- and post-purification S binding titers (Fig. 1e; *R* = 0.8215, *P* < 0.0001), RBD binding titers (Fig. 1f; *R* = 0.7721, *P* < 0.0001), and pseudovirus neutralizing antibody titers (Fig. 1g; *R* = 0.8252, *P* < 0.0001), demonstrating that the purified IgG retained the binding and neutralizing capacity of original plasma samples.

### Passive transfer of IgG to naïve hamster recipients

We then assessed the protective efficacy of these purified IgG stocks in an adoptive transfer study in golden Syrian hamsters. For each study participant, 4–5 hamsters received an intra-peritoneal injection of 25 mg of purified IgG. Experimental controls included groups of hamsters that received buffer alone as negative controls (*n* = 15) or 25 mg of IgG purified from convalescent re-challenged non-human primates (NHPs) after two rounds of SARS-CoV-2 challenge^8^ as high-titer positive controls (*n* = 11).

One day after administration of IgG or buffer control, serum was collected from all hamsters to quantify post-transfer, pre-challenge SARS-CoV-2 spike-binding and neutralizing antibody titers. As expected, low or undetectable binding antibody titers to S (Fig. 1h, Supplementary Fig. 1) and RBD (Fig. 1i, Supplementary Fig. 1) proteins were observed in hamsters administered with IgG from placebo recipients, as well as in hamsters that received the buffer control (Supplementary Fig. 1). In contrast, detectable binding titers were observed in all groups of hamsters that received IgG from vaccine recipients, with median titers of 2.8 × 10^3^ and 1.2 × 10^3^ for S and RBD binding, respectively. Similarly, low or undetectable neutralizing titers were observed in groups of hamsters administered IgG from placebo recipients or buffer only control (Fig. 1j, Supplementary Fig. 1). In groups of hamsters that received IgG from vaccine recipients, detectable neutralizing titers were observed in groups corresponding to 12/20 participants (Fig. 1j). Post-transfer, the median neutralizing titers in hamster serum ranged from 20 to 121, with a median value of 27, values approximately 10-fold lower than the neutralizing titers measured in corresponding human plasma, as a result of the dilution following adoptive transfer. Further, transfer of high-titer positive control IgG from convalescent re-challenged NHPs resulted in high post-transfer neutralizing activity, with a median hamster serum titer of 264 (Supplementary Fig. 1). Strong positive correlations were observed between the binding and neutralizing activity in hamster serum post-transfer, supporting the exploration of these parameters as potential immune correlates of protection following challenge (Supplementary Fig. 2).

### SARS-CoV-2 challenge and monitoring of clinical disease

**Fig. 2:**
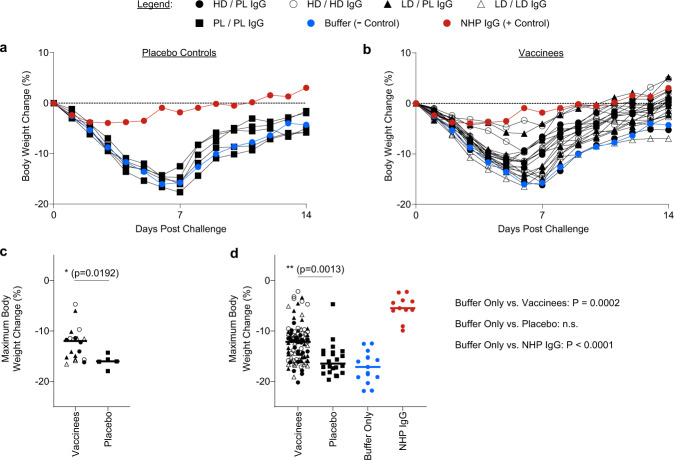
IgG purified from human Ad26.COV2.S vaccine recipients attenuates SARS-CoV-2-associated clinical disease in hamsters. One day post-IgG transfer, groups of hamsters were challenged with WA1/2020 SARS-CoV-2 via the intranasal route. Post-challenge, hamsters were monitored for fourteen days for signs of clinical disease (i.e., weight loss). **a** Data shown represent the median body weight change for the following groups: negative control (buffer only), positive control (NHP IgG), or each of the *N* = 5 clinical trial study participants that received a placebo immunization. **b** Data shown represent the median of the groups corresponding to the *N* = 20 participants who received Ad26.COV2.S vaccine at the indicated dosing regimens. For reference, the buffer only and NHP IgG data are reproduced in panel b to allow for comparison of experimental groups with negative and positive controls. **c**, **d** The maximum body weight change was calculated for each hamster in the study. Data points displayed in panel **c** correspond to the group median maximum body weight change for each group of hamsters that corresponds to one study participant. Horizontal lines indicate the group medians and statistics indicated are the results of a two-sided Mann-Whitney test, comparing vaccinees to placebo controls. In panel **d**, maximum body weight change is displayed for each individual hamster receiving IgG from Ad26.COV2.S vaccinees, placebo control IgG, or the control groups of buffer only or convalescent NHP IgG. Horizontal lines indicate medians. Statistics displayed are the results of a Kruskal-Wallis test with Dunn’s multiple comparisons test; for clarity, the comparison of vaccinees vs. placebo is indicated on the plot, while the p values comparing the buffer only control to the indicated groups are displayed next to the plot.

All recipient hamsters were then challenged with 2 × 10^4^ TCID_50_ of WA1/2020 SARS-CoV-2 via the intra-nasal route. Hamsters were monitored for fourteen days post-challenge for body weight loss. In the negative control group (hamsters receiving buffer only), animals exhibited significant weight loss over approximately seven days, followed by a gradual recovery by day 14 (Fig. 2a, Supplementary Fig 3). Similarly, in hamsters that received IgG purified from placebo recipients, comparable severe weight loss was observed (Fig. 2a, Supplementary Fig. 3). In contrast, hamsters that received convalescent NHP IgG exhibited attenuated weight loss (Fig. 2b, Supplementary Fig. 3).

To quantitate protective efficacy across experimental groups, the maximum body weight change was calculated for each group of hamsters, each of which corresponded to one human vaccine recipient. The median peak body weight loss of placebo recipients (−16.0%) was greater than vaccine recipients (−12.0%) (Fig. 2c, *P* = 0.0192, Mann–Whitney test). Similar analyses were conducted analyzing the peak weight loss of each individual hamster (Fig. 2d, Supplementary Fig, 4). In hamsters that received buffer only, median maximum weight loss was 17.1%. In groups that received IgG from placebo recipients, no significant impact was observed with a median maximum weight loss of 16.5% (*P* > 0.9999, Kruskal–Wallis test with Dunn’s correction for multiple comparisons). In contrast, recipients of the positive control high-titer IgG from convalescent, re-challenged NHPs exhibited significantly reduced peak weight loss of 5.5% (*P* < 0.0001, Kruskal–Wallis test with Dunn’s correction for multiple comparisons). Moreover, in groups that received IgG purified from Ad26.COV2.S recipients, reduced median peak weight loss of 12.2% was observed (*P* = 0.0002, Kruskal–Wallis test with Dunn’s correction for multiple comparisons).

### Analyses of immune correlates of protection

**Fig. 3:**
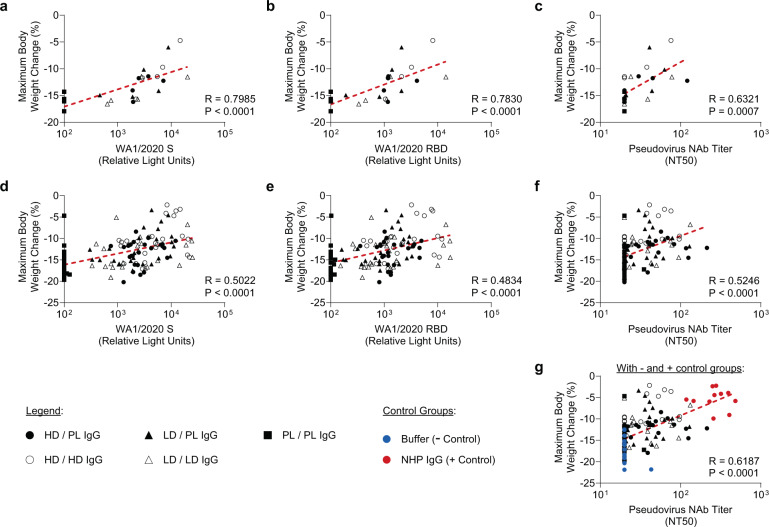
Post-transfer binding and neutralizing antibody activity correlate with post-challenge body weight loss. Correlation analyses were performed between post-transfer, pre-challenge binding and neutralizing antibody activity and the maximum body weight change following WA1/2020 SARS-CoV-2 challenge. In panels **a**–**c**, the median (**a**) WA1/2020 spike (S) or (**b**) WA1/2020 receptor binding domain (RBD) titer or (**c**) the median WA1/2020 pseudovirus neutralization titer was correlated with the median maximum body weight change. Data points displayed correspond to the median values for each group of hamsters corresponding to one study participant. To enable visualization of the full data set, panels **d**–**g** display similar analyses, with data points corresponding to all individual hamsters in the study shown. Median maximum body weight change was correlated with (**d**) WA1/2020 S binding, (**e**) WA1/2020 RBD binding, and (**f**) WA1/2020 pseudovirus NT50; data is displayed for all hamsters that received IgG from a vaccinee or placebo control. In panel (**g**) the buffer only and NHP IgG groups are also displayed to enable visualization of the experimental data set overlaid with negative and positive controls. Statistics displayed in all panels indicate the results of Spearman correlation analyses.

To characterize the correlation between antibody titer and protective efficacy, a correlation analysis was performed between pre-challenge serum SARS-CoV-2 spike binding or neutralizing titers in the recipient hamsters and post-challenge maximum body weight change (Fig. 3a–g). When analyzing the median values of each hamster group corresponding to one clinical trial participant, significant correlations were observed between body weight loss and S binding (Fig. 3a; *R* = 0.7985, *P* < 0.0001), RBD binding (Fig. 3b; *R* = 0.7830, *P* < 0.0001), and psedudovirus neutralizing (Fig. 3c; *R* = 0.6321, *P* = 0.0007) antibody titers. Significant correlations were also observed through similar analyses of each individual hamster between peak weight loss and S binding (Fig. 3d; *R* = 0.5022, *P* < 0.0001) or RBD binding (Fig. 3e; *R* = 0.4834, *P* < 0.0001) and neutralizing (Fig. 3f; *R* = 0.5246, *P* < 0.0001) antibody titers. An additional analysis confirmed that inclusion of the control groups generated similar correlations (Fig. 3g, *R* = 0.6187, *P* < 0.0001). Together, these data demonstrate that vaccine-elicited SARS-CoV-2-specific spike binding and neutralizing antibody titers correlated with protection against SARS-CoV-2 challenge following adoptive transfer in hamsters.

## Discussion

Our data demonstrate that passive transfer of purified polyclonal IgG from Ad26.COV2.S vaccinated humans confers protection from weight loss following high-dose SARS-CoV-2 challnege in hamsters. These data demonstrate directly and mechanistically that SARS-CoV-2-specific binding and neutralizing antibodies from vaccinated humans have protective capacity. While previous studies have revealed that antibody titers, particularly neutralizing antibody titers, are statistical correlates of protection^1,2,8,16^, study of the function of vaccine-elicited antibodies from humans remains an underexplored question. This approach has previously been applied for vaccine development for Zika virus^17,18^ and other pathogens to isolate and study the role of vaccine-elicited antibodies from human clinical trial participants. Further, in the context of the COVID-19 pandemic, enhanced understanding of key immune signatures that underscore protection could enable evaluation of novel candidate vaccines in the absence of large-scale placebo-controlled clinical trials^16,19^.

In this study, a high-dose challenge in golden Syrian hamsters was employed to model severe clinical disease. Towards this goal, hamsters were monitored for two weeks post-challenge for weight loss, limiting the potential to analyze viral loads in tissues at the time of peak viral replication. Future studies could be designed to focus more directly on the potential for transferred IgG to drive virologic control in respiratory tract tissues. The stringency of this challenge model, together with the 10-fold dilution of antibody titers following adoptive transfer, likely explain the incomplete protection observed. Moreover, adoptive transfer of purified IgG excludes the potential contribution of T cell responses to protection. In addition, anamnestic responses are absent in the adoptive transfer model, requiring antibodies at the time of challenge to protect against severe disease without immunologic memory recall responses.

Protection correlated with pre-challenge binding and neutralizing titers post-IgG transfer. Confirmation of such an antibody correlate of protetion can support the development and characterization of new vaccines or antibody-based therapeutics, as well as inform public health strategies^19^. However, other immunologic parameters, including Fc functional antibodies, cellular immune responses, and innate parameters may also contribute to protection. Our findings that antibody titers mediate protection against severe diesease is consistent with recently-reported large-scale Ad26.COV2.S efficacy trials^14^. These data revealed significant protection from severe COVID-19, with efficacy of 86%, 88%, and 82% in the United States, Brazil, and South Africa, respectively^14^.

Taken together, our data demonstrate that binding and neutralizing antibodies following Ad26.COV2.S vaccination in humans directly contribute to protective efficacy against SARS-CoV-2 in a titer-dependent fashion in a preclinical adoptive transfer model. However, Fc-mediated activity of vaccine induced antibodies may be absent when human IgG is transferred to hamster recipients^13^, and T cell responses have also been reported to contribute to protection in pre-clinical and clinical studies^19,20^. Future studies could explore the role of Ad26.COV2.S-elicited T cell responses or other aspects of the adaptive immune response (e.g., IgG subclass, isotype) in mediating protection against SARS-CoV-2 infection and disease.

## Methods

### Clinical study design and human plasma samples

Plasma samples were collected from participants in Cohort 1b of as a part of the Phase 1/2a clinical trial of Ad26.COV2.S vaccine (NCT04436276)^12^. This study was conducted at a single site at Beth Israel Deaconess Medical Center in Boston with review and approval by the Beth Israel Deaconess Medical Center institutional review board. All participants gave written informed consent and successfully completed an assessment of understanding before the initiation of study procedures. This descriptive study was performed as part of a larger multicenter, randomized, double-blind, placebo-controlled phase 1/2a trial to evaluate the safety, reactogenicity, and immunogenicity of Ad26.COV2.S at 5 × 10^10^ or 1 × 10^11^ viral particle doses. Vaccines was administered intramuscularly as either a single-shot or 2-shot vaccine schedules, 56 days apart, in healthy adults. Cohort 1b enrolled adults 18 to 55 years of age (*N* = 25).

### IgG purification

Polyclonal IgG was purified from human plasma samples collected on day 71, two weeks after the second immunization of either Ad26.COV2.S or placebo. This purification was conducted using NAb Protein G spin columns (Thermo Scientific). Protein G columns were preconditioned with Protein G IgG Binding Buffer (Thermo Scientific) and plasma samples were added to columns and incubated for 1 h. Columns were then washed, followed by elution with IgG Elution Buffer (Thermo Scientific). Eluted, purified IgG was buffer exchanged with 1 x DPBS and characterized by SDS-PAGE and spectrophotometry was used to quantify yield and normalize stocks to 10 mg-ml^−1^.

### Electrochemiluminescence assay (ECLA)

ECLA plates to quantify binding antibody responses to WA1/2020 spike (S) and Receptor Binding Domain (RBD) proteins were selected (MesoScale Discovery SARS-CoV-2 IgG Cat No: N05CA-1; Panel 11, 13)^13^. Plates were blocked with 50 uL of Blocker A (1% BSA in distilled water) for 30 m at room temperature shaking at 700 rpm with a digital microplate shaker. During blocking the serum was diluted 1:5,000 in Diluent 100. The plates were then washed 3 times with 150 μL of wash buffer (0.5% Tween in 1x PBS) per well, blotted dry, and 50 μL of the pre-diluted samples were added in duplicate to the plates and set to shake at 700 rpm at room temperature for at least 2 h. The plates were again washed 3 times and 50 µl of SULFO-Tagged anti-Human IgG detection antibody was added to each welwas added to each well and incubated shaking at 700 rpm at room temperature for at least 1 h. Plates were then washed 3 times and 150 μL of MSD GOLD Read Buffer B was added to each well and the plates were read immediately after on a MESO QuickPlex SQ 120 machine. MSD titers for each sample was reported as Relative Light Units (RLU) which were calculated as average Sample RLU minus Blank RLU for each sample. The limit of detection was defined as 100 RLU for each assay.

### Pseudovirus neutralization assay

A SARS-CoV-2 pseudovirus, incorporating a luciferase reporter gene, was generated similar to previously described approaches^1,2,21^. To generate pseudoviruses, HEK293T cells were co-transfected with packaging construct psPAX2 (AIDS Resource and Reagent Program), a luciferase reporter plasmid pLenti-CMV Puro-Luc (Addgene), and a plasmid expressing S protein, pcDNA3.1-SARS-CoV-2 SΔCT with lipofectamine 2000. 48 h after transfection, supernatants containing pseudoviruses were collected and filtered through a 0.45-µm filter. To quantify neutralizing activity of human plasma samples or purified IgG stocks, HEK293T target cells, transfected to express human ACE2 (HEK293T-hACE2), were seeded in 96-well tissue culture plates at a density of 1.75 × 10^4^ cells per well and cultured overnight. Three-fold serial dilutions of heat-inactivated plasma or purified IgG samples were prepared, mixed with 50 µl of pseudovirus, incubated for 1 h at 37 °C, and then added to HEK293T-hACE2 seeded wells. After 48 h of incubation, cells were lysed in Steady-Glo Luciferase Assay Reagent (Promega) according to the manufacturer’s instructions. SARS-CoV-2 neutralization titers were defined as the sample dilution at which a 50% reduction in relative light units was observed relative to the average of the control wells treated with virus only.

### Hamster study design

One-hundred and forty-six male and female Syrian golden hamsters (Envigo), 10–12 weeks old, were randomly allocated to groups. On day −1, animals received an intra-peritoneal injection containing either 25 mg of purified IgG in 2.5 mL of buffer, or 2.5 mL of buffer alone. On day 0, blood was collected via the retro-orbital route to quantify post-transfer serum neutralizing antibody titers. Immediately following blood draw, all animals were challenged with 1.99 × 10^4^ TCID_50_ SARS-CoV-2, which was derived with one passage from USA-WA1/2020 (NR- 53780, BEI Resources). Virus was administered as 100 μl by the intranasal route (50 μl in each nare). Body weights were assessed daily. Weight loss that exceeded 20% of body weight on the day of challenge was established as a humane endpoint euthanasia criteria. Due to the large N, the study was broken down into three cohorts, with a negative control (i.e., buffer only) and a positive control (convalescent NHP IgG) group included in each of the three experiments. Data presented for the control groups are pooled from the three independent challenge studies, which each exhibited similar results.

### Ethical statement

All animal studies were conducted in compliance with all relevant local, state, and federal regulations. All experiments were approved by the Bioqual Institutional Animal Care and Use Committee (IACUC).

### Reporting Summary

Further information on research design is available in the Nature Research Reporting Summary linked to this article.

## Supplementary information

Supplementary Information

Reporting Summary

## Data Availability

All data are available in the main text or the supplementary materials.
