# Supplementary material for: Passive transfer of Ad26.COV2.S-elicited IgG from humans attenuates SARS-CoV-2 disease in hamsters
Source: NPJ Vaccines. 2022 Jan 10;7:2. doi: 10.1038/s41541-021-00427-z (PMC8748674; doi:10.1038/s41541-021-00427-z)
Supplement: Supplementary file 1 — Supplementary Information [file 41541_2021_427_MOESM1_ESM.pdf]

Supplementary Information

Passive transfer of Ad26.COV2.S-elicited IgG from humans attenuates SARS-CoV-2 disease in hamsters

Lisa H. Tostanoski[1], Abishek Chandrashekar[1], Shivani Patel[1], Jingyou Yu[1], Catherine Jacob-Dolan[1,2], Aiquan Chang[1,2], Olivia C. Powers[1], Daniel Sellers[1], Sarah Gardner[1], Julia Barrett[1], Owen Sanborn[1],Kathryn E. Stephenson[1], Jessica L. Ansel[1], Kate Jaegle[1], Michael S. Seaman[1], Maciel Porto[3], Megan Lok[3], Brittany Spence[3], Kathleen Cayer[3], Danielle Nase[3], Shaikim Holman[3], Heath Bradette[3], Swagata Kar[3], Hanne Andersen[3], Mark G. Lewis[3], Freek Cox[4], Jeroen T. B. M. Tolboom[4], Anne Marit de Groot[4], Dirk Heerwegh[5], Mathieu Le Gars[4], Jerald Sadoff[4], Frank Wegmann[4], Roland C. Zahn[4], Hanneke Schuitemaker[4], Dan H. Barouch[1,2,6,7*]

[1]Center for Virology and Vaccine Research, Beth Israel Deaconess Medical Center, Harvard Medical School; Boston, MA, USA.

[2]Harvard Medical School; Boston, MA, USA.

[3]BIOQUAL, Inc.; Rockville, MD, USA.

[4]Janssen Vaccines & Prevention, Leiden, The Netherlands.

[5]Janssen Research & Development, Beerse, Belgium

[6]Ragon Institute of MGH, MIT and Harvard; Cambridge, MA, USA.

[7]Massachusetts Consortium on Pathogen Readiness, Boston, MA, USA.

*Corresponding author. Email: dbarouch@bidmc.harvard.edu

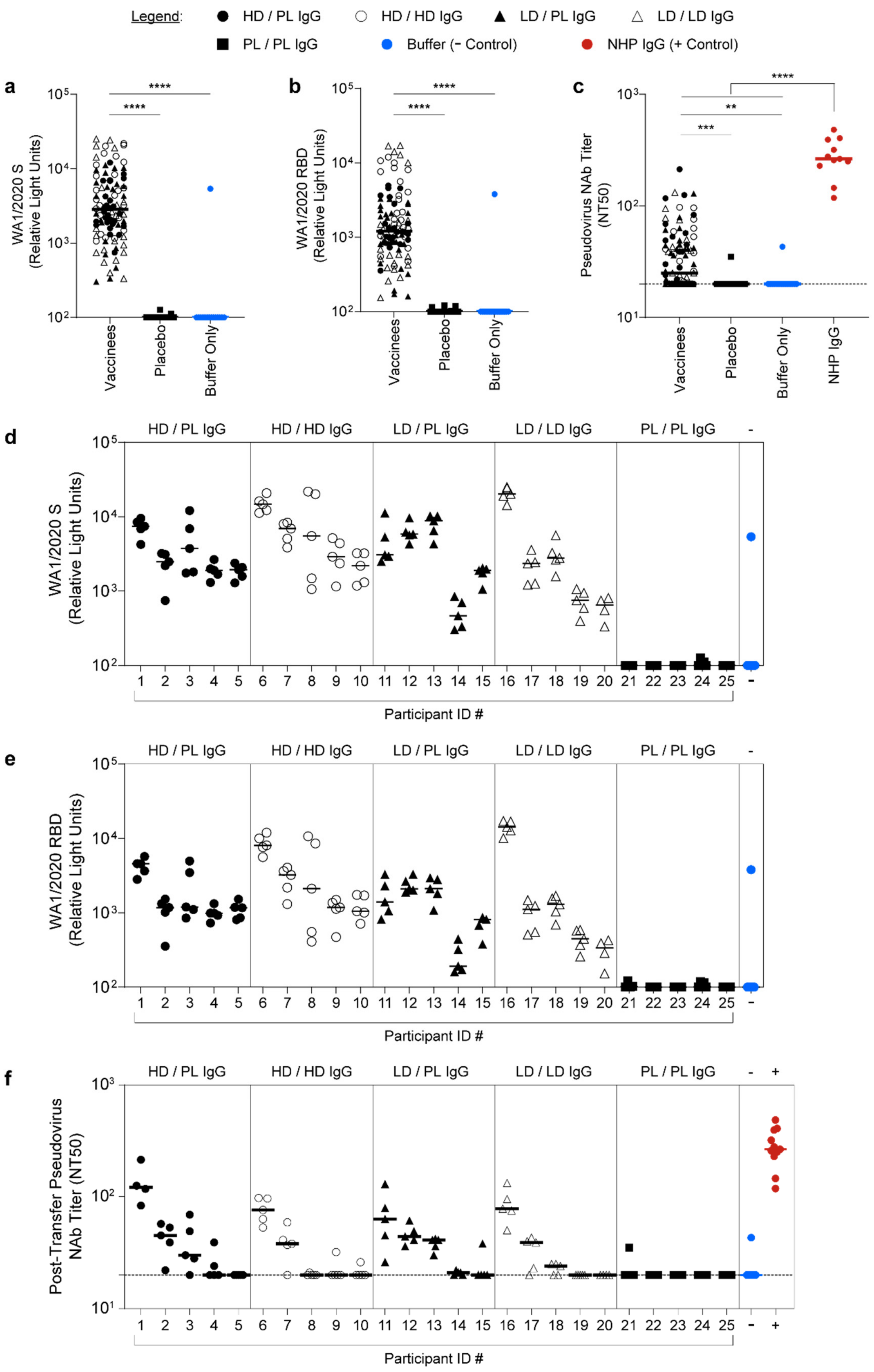
Legend:
HD / PL IgG
HD / HD IgG
LD / PL IgG
LD / LD IgG
PL / PL IgG
Buffer (- Control)
NHP IgG (+ Control)
a
WA1/2020 S (Relative Light Units)
Vaccinees
Placebo
Buffer Only
b
WA1/2020 RBD (Relative Light Units)
c
Pseudovirus NAb Titer (NT50)
NHP IgG
d
e
f
Post-Transfer Pseudovirus NAb Titer (NT50)
Participant ID #

Supplementary Figure 1. Purified IgG from vaccine or placebo recipients was transferred to naïve hamsters via intraperitoneal injection and one-day post-transfer, serum was analyzed for **a)** WA1/2020 SARS-CoV-2 spike (S) binding, **b)** WA1/2020 SARS-CoV-2 receptor binding domain (RBD) binding, or **c)** WA1/2020 pseudovirus neutralization titers. Additional control groups included hamsters that received buffer alone and hamsters that received an equivalent dose (i.e., 25 mg) of IgG purified from convalescent non-human primates (NHPs). Data displayed in a-b were generated via electrochemiluminescence assays (ECLA) using an anti-human detection antibody, thus the NHP IgG recipient group was excluded from the analyses. In panels a-c, data points displayed correspond to the value of each individual recipient hamsters and horizontal lines indicate group medians. Statistics displayed are the results of Kruskal-Wallis tests with Dunn's multiple comparisons test. (** = $P \leq 0.01$; *** = $P \leq 0.001$; **** = $P \leq 0.0001$). Similarly, in panels **d-f)** the binding and neutralizing antibody titers of individual hamsters are displayed, separated into groups corresponding to the N=20 study participants who received the Ad26.COV2.S vaccine, the N=5 participants who received a placebo immunization, or the control hamsters that received either buffer alone or convalescent NHP IgG. Within each dosing regimen, particpants were ordered and assigned a number 1-25 by magnitude of response for visualization purposes. As above, in panels d-e, the NHP IgG recipient group was excluded from ECLA analyses due to the human-specific detection antibody. Horizontal lines in panels d-f indicate the group medians.

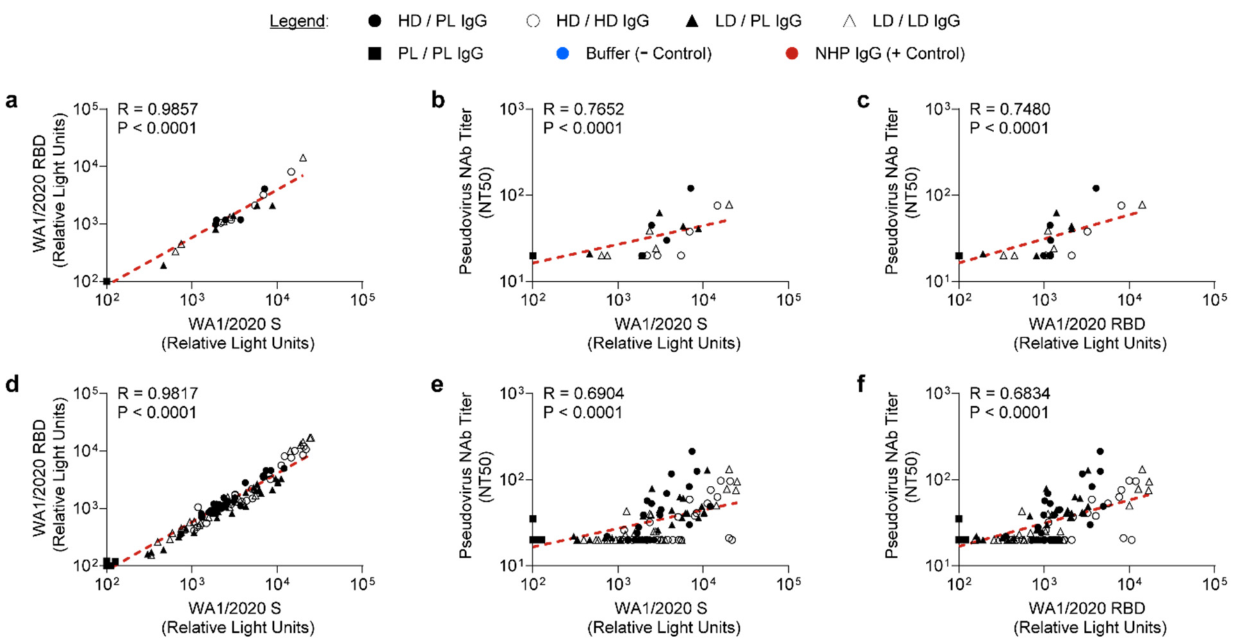

Supplementary Figure 2. Post-transfer binding and neutralizing antibody activity correlation analyses. Purified IgG from vaccine or placebo recipients was transferred to naïve hamsters via intraperitoneal injection and one-day post-transfer, serum was analyzed for **a)** WA1/2020 SARS-CoV-2 spike (S) binding, **b)** WA1/2020 SARS-CoV-2 receptor binding domain (RBD) binding, or **c)** WA1/2020 pseudovirus neutralization titers. Correlation analyses between these three assays were completed for the a-c) median values for each group of hamsters corresponding to one study participant and d-f) each individual hamster as a single data point. Statistics in all panels indicate the results of Spearman correlation analyses.

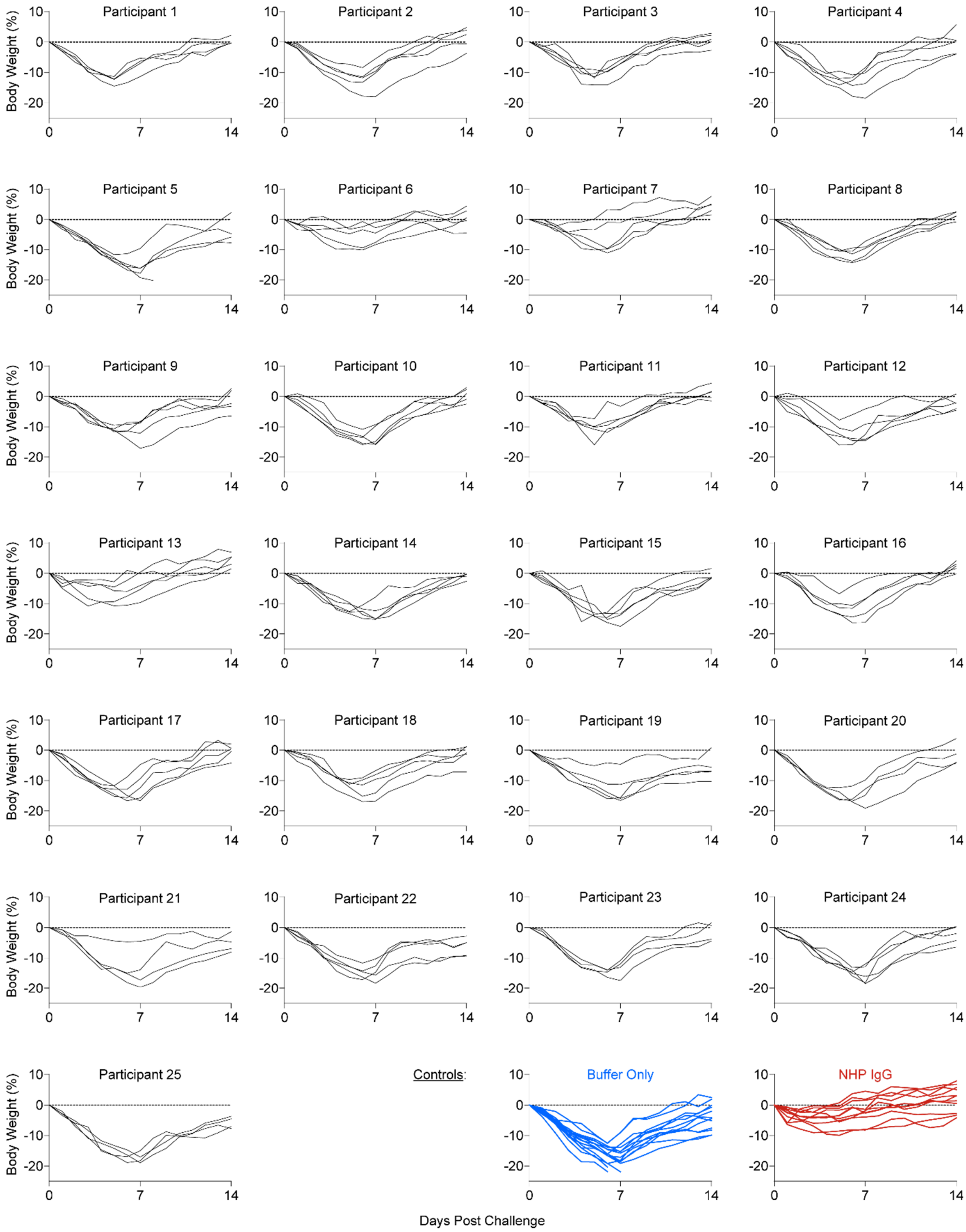

Participant 1
Participant 2
Participant 3
Participant 4
Participant 5
Participant 6
Participant 7
Participant 8
Participant 9
Participant 10
Participant 11
Participant 12
Participant 13
Participant 14
Participant 15
Participant 16
Participant 17
Participant 18
Participant 19
Participant 20
Participant 21
Participant 22
Participant 23
Participant 24
Participant 25
Controls:
Buffer Only
NHP IgG
Body Weight (%)
Days Post Challenge

Supplementary Figure 3. One day post-IgG transfer, groups of hamsters were challenged with SARS-CoV-2 via the intranasal route. Post-challenge, hamsters were monitored for fourteen days for signs of clinical disease. Data shown represent the individual weight traces of each hamster in the groups corresponding to the study participants (N=25), as well as the negative control (buffer only) and positive control (NHP IgG) groups. Study participants received the following dosing regimens, as described in Table 1: i) Participants 1-5: HD/PL, Participants 6-10: HD/HD, Participants 11-15: LD/PL, Participants 16-20: LD/LD, and Participants 21-25: PL/PL. Incomplete body weight traces represent animals that exhibited greater than 20% body weight loss, a humane endpoint euthanasia criteria.

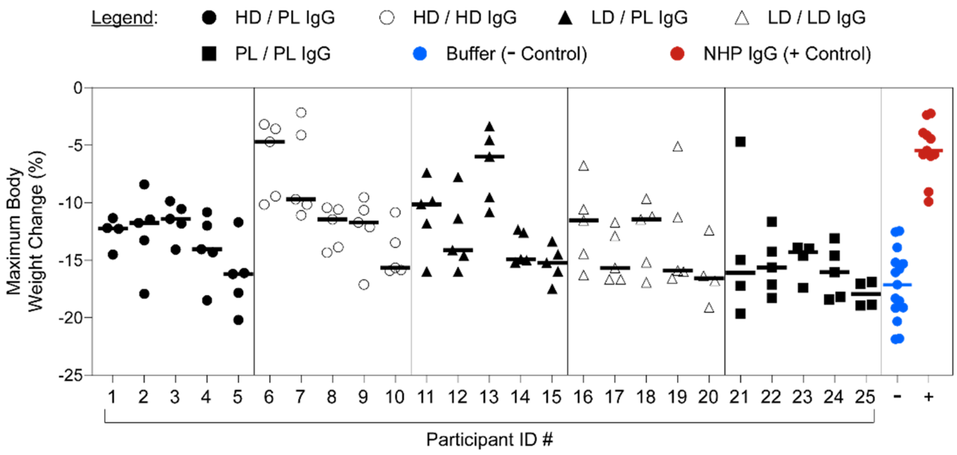

Supplementary Figure 4. Data displayed correspond to the median maximum body weight change, similar to the data displayed in Fig 2c-d. In these Supplementary, maximum body weight change is shown for each individual hamster in the groups corresponding to the study participants or the control groups, as indicated

Supplementary Table 1. Study overview of the Ad26.COV2.S dosing regimens tested in cohort 1b.

Group	N	Day 1	Day 57	Code
1	5	1 x 10^{11} VP Ad26.COV2.S	Placebo	HD / PL
2	5	1 x 10^{11} VP Ad26.COV2.S	1 x 10^{11} VP Ad26.COV2.S	HD / HD
3	5	5 x 10^{10} VP Ad26.COV2.S	Placebo	LD / PL
4	5	5 x 10^{10} VP Ad26.COV2.S	5 x 10^{10} VP Ad26.COV2.S	LD / LD
5	5	Placebo	Placebo	PL / PL
