# Supplementary material for: Passive transfer of Ad26.COV2.S-elicited IgG from humans attenuates SARS-CoV-2 disease in hamsters
Source: NPJ Vaccines. 2022 Jan 10;7:2. doi: 10.1038/s41541-021-00427-z (PMC8748674; doi:10.1038/s41541-021-00427-z)
Supplement: Supplementary file 2 — Reporting Summary [file 41541_2021_427_MOESM2_ESM.pdf]

nature portfolio

Corresponding author(s): Dan H. Barouch

Last updated by author(s): November 28, 2021

Reporting Summary

Nature Portfolio wishes to improve the reproducibility of the work that we publish. This form provides structure for consistency and transparency in reporting. For further information on Nature Portfolio policies, see our Editorial Policies and the Editorial Policy Checklist.

Statistics

For all statistical analyses, confirm that the following items are present in the figure legend, table legend, main text, or Methods section.

n/a	Confirmed	
☐	☒	The exact sample size (*n*) for each experimental group/condition, given as a discrete number and unit of measurement
☐	☒	A statement on whether measurements were taken from distinct samples or whether the same sample was measured repeatedly
☐	☒	The statistical test(s) used AND whether they are one- or two-sided<br>*Only common tests should be described solely by name; describe more complex techniques in the Methods section.*
☒	☐	A description of all covariates tested
☐	☒	A description of any assumptions or corrections, such as tests of normality and adjustment for multiple comparisons
☐	☒	A full description of the statistical parameters including central tendency (e.g. means) or other basic estimates (e.g. regression coefficient) AND variation (e.g. standard deviation) or associated estimates of uncertainty (e.g. confidence intervals)
☐	☒	For null hypothesis testing, the test statistic (e.g. *F*, *t*, *r*) with confidence intervals, effect sizes, degrees of freedom and *P* value noted<br>*Give P values as exact values whenever suitable.*
☒	☐	For Bayesian analysis, information on the choice of priors and Markov chain Monte Carlo settings
☒	☐	For hierarchical and complex designs, identification of the appropriate level for tests and full reporting of outcomes
☒	☐	Estimates of effect sizes (e.g. Cohen's *d*, Pearson's *r*), indicating how they were calculated

Our web collection on statistics for biologists contains articles on many of the points above.

Software and code

Policy information about availability of computer code

Data collection	N/A
Data analysis	Microsoft Excel 2013, GraphPad Prism 9.2.0

For manuscripts utilizing custom algorithms or software that are central to the research but not yet described in published literature, software must be made available to editors and reviewers. We strongly encourage code deposition in a community repository (e.g. GitHub). See the Nature Portfolio guidelines for submitting code & software for further information.

Data

Policy information about availability of data

All manuscripts must include a data availability statement. This statement should provide the following information, where applicable:

- Accession codes, unique identifiers, or web links for publicly available datasets
- A description of any restrictions on data availability
- For clinical datasets or third party data, please ensure that the statement adheres to our policy

Data availability statement included. All data are available in the manuscript and supplementary information.

Field-specific reporting

Please select the one below that is the best fit for your research. If you are not sure, read the appropriate sections before making your selection.

☒ Life sciences ☐ Behavioural & social sciences ☐ Ecological, evolutionary & environmental sciences

For a reference copy of the document with all sections, see nature.com/documents/nr-reporting-summary-flat.pdf

Life sciences study design

All studies must disclose on these points even when the disclosure is negative.

Sample size	Sample size was 25 individuals (5 individuals/group reflecting 20 vaccine recipients and 5 placebo recipients). Details provided in the primary clinical trials manuscript (Stephenson et al. JAMA 2021; 325:1535-1544). For hamster recipients, group sizes of N=4-5 per clinical trial participant were used to characterize impact on clinical disease following challenge.
Data exclusions	One hamster was excluded from the study. A health issue (eye injury) was discovered during the study that required euthanasia per veterinary recommendation. Due to incomplete weight loss curve data, this animal was omitted from the experiment.
Replication	Immunologic assays (ECLA) were performed in duplicate. Technical replicates were similar. Attempts at replication were successful; for example, three independent cohorts of negative control (buffer only) and positive control (convalenscent NHP IgG) were studied and found to exhibit similar immunologic results and challenge-induced weight loss.
Randomization	Participants in COV1001 cohort 1b were randomly allocated to groups. Hamster recipients were randomly allocated to groups.
Blinding	COV1001 was a double-blinded study. All immunologic and virologic assays were also performed blinded.

Reporting for specific materials, systems and methods

We require information from authors about some types of materials, experimental systems and methods used in many studies. Here, indicate whether each material, system or method listed is relevant to your study. If you are not sure if a list item applies to your research, read the appropriate section before selecting a response.

Materials & experimental systems

n/a	Involved in the study	
☒	☐	Antibodies
☐	☒	Eukaryotic cell lines
☒	☐	Palaeontology and archaeology
☐	☒	Animals and other organisms
☐	☒	Human research participants
☐	☒	Clinical data
☒	☐	Dual use research of concern

Methods

n/a	Involved in the study	
☒	☐	ChIP-seq
☒	☐	Flow cytometry
☒	☐	MRI-based neuroimaging

Eukaryotic cell lines

Policy information about cell lines

Cell line source(s)	HEK293T from ATCC
Authentication	Commercially purchased (ATCC) and evaluated in control experiments prior to use.
Mycoplasma contamination	Negative for mycoplasma
Commonly misidentified lines (See ICLAC register)	N/A

Animals and other organisms

Policy information about studies involving animals; ARRIVE guidelines recommended for reporting animal research

Laboratory animals	Male and female Syrian golden hamsters (Envigo), 10–12 weeks old were utilized in the study.
Wild animals	N/A

Field-collected samples	N/A
Ethics oversight	All animal studies were conducted in compliance with all relevant local, state and federal regulations and were approved by the Bioqual Institutional Animal Care and Use Committee.

Note that full information on the approval of the study protocol must also be provided in the manuscript.

Human research participants

Policy information about studies involving human research participants

Population characteristics	Healthy adults 18-55, mixed gender; details provided in the primary clinical trial manuscript (Stephenson et al., JAMA 2021; 325:1535-1544)
Recruitment	Participants were recruited by education and outreach programs; details provided in the primary clinical trial manuscript (Stephenson et al., JAMA 2021; 325:1535-1544)
Ethics oversight	BIDMC Institutional Review Board; details provided in the primary clinical trial manuscript (Stephenson et al., JAMA 2021; 325:1535-1544)

Note that full information on the approval of the study protocol must also be provided in the manuscript.

Clinical data

Policy information about clinical studies

All manuscripts should comply with the ICMJE guidelines for publication of clinical research and a completed CONSORT checklist must be included with all submissions.

Clinical trial registration	NCT04436276
Study protocol	Primary clinical trial and protocol published (Stephenson et al., JAMA 2021; 325:1535-1544)
Data collection	25 participants were enrolled from July 29, 2020 to August 7, 2020 and were followed through day 71 (completed on October 3, 2020). This study was conducted at a single clinical site in Boston, MA as part of a randomized, double-blinded, placebo-controlled phase 1/2a clinical trial (COV1001) of Ad26.COV2.S
Outcomes	Safety and immunogenicity are reported in the primary clinical trial manuscript (Stephenson et al., JAMA 2021; 325:1535-1544)
